# Quantifying population-specific growth in benthic bacterial communities under low oxygen using H_2_^18^O

**DOI:** 10.1038/s41396-019-0373-4

**Published:** 2019-02-19

**Authors:** Ömer K. Coskun, Volkan Özen, Scott D. Wankel, William D. Orsi

**Affiliations:** 1 Department of Earth and Environmental Sciences, Paleontology and Geobiology, Ludwig-Maximilians-Universität München, 80333 Munich, Germany; 2 Department of Marine Chemistry and Geochemistry, Woods Hole Oceanographic Institution, Woods Hole, MA 02543 USA; 3 GeoBio-Center, Ludwig-Maximilians-Universität München, 80333 Munich, Germany

**Keywords:** Microbial ecology, Population dynamics, Microbiome, Marine microbiology, Bacterial physiology

## Abstract

The benthos in estuarine environments often experiences periods of regularly occurring hypoxic and anoxic conditions, dramatically impacting biogeochemical cycles. How oxygen depletion affects the growth of specific uncultivated microbial populations within these diverse benthic communities, however, remains poorly understood. Here, we applied H_2_^18^O quantitative stable isotope probing (qSIP) in order to quantify the growth of diverse, uncultured bacterial populations in response to low oxygen concentrations in estuarine sediments. Over the course of 7- and 28-day incubations with redox conditions spanning from hypoxia to euxinia (sulfidic), ^18^O labeling of bacterial populations exhibited different patterns consistent with micro-aerophilic, anaerobic, facultative anaerobic, and aerotolerant anaerobic growth. ^18^O-labeled populations displaying anaerobic growth had a significantly non-random phylogenetic distribution, exhibited by numerous clades currently lacking cultured representatives within the *Planctomycetes*, *Actinobacteria*, *Latescibacteria*, *Verrucomicrobia*, and *Acidobacteria*. Genes encoding the beta-subunit of the dissimilatory sulfate reductase (dsrB) became ^18^O labeled only during euxinic conditions. Sequencing of these ^18^O-labeled *dsrB* genes showed that *Acidobacteria* were the dominant group of growing sulfate-reducing bacteria, highlighting their importance for sulfur cycling in estuarine sediments. Our findings provide the first experimental constraints on the redox conditions underlying increased growth in several groups of “microbial dark matter”, validating hypotheses put forth by earlier metagenomic studies.

## Introduction

Benthic microbial communities living in estuarine ecosystems play an important role in global biogeochemical cycles, because they drive organic matter decomposition, nutrient regeneration, and influence water column dissolved O_2_ concentrations [1, 2]. Benthic oxygen depletion is typical in estuarine habitats, where degradation of organic matter is 100–1000 times higher than corresponding values in the water column [3]. The resulting hypoxia impacts both fisheries by increasing fish mortality [4], as well as climate by facilitating increased fluxes of the greenhouse gas nitrous oxide [5].

Estuarine sediments have complex microbial communities composed primarily of uncultured lineages catalyzing aerobic, micro-aerophilic, and anaerobic metabolic pathways that impact carbon, nitrogen, and sulfur cycling [6]. Quantifying growth of specific bacterial populations is challenging, yet critical for understanding of ecosystem resilience and response to change [7]. The structure of microbial communities in estuarine habitats can exhibit resistance to environmental perturbation [8], but the levels of activity within populations can change dramatically in response to changing nutrients and oxygen levels, with clear impacts on biogeochemical cycles [9].

The activity of growing microbial populations in environmental samples can be quantified using quantitative DNA-stable isotope probing (qSIP) with H_2_^18^O as a passive tracer [10]. Oxygen atoms from H_2_^18^O are incorporated into DNA during genome replication, which when combined with quantitative PCR (qPCR) and high-throughput sequencing of 16S rRNA genes can be used to quantify activity of growing populations within complex microbial communities from environmental samples [11, 12]. Relative to energy-rich ^13^C-labeled carbon substrates, labeled water is a passive tracer of cell growth, whereby DNA replication generates a new DNA strand that will contain ^18^O atoms in the presence of labeled water [12]. The amount of ^18^O incorporated into the total DNA pool is correlated with growth rates [11–13], showing that ^18^O labeling occurs primarily during growth via DNA replication [12]. The degree of atomic incorporation can then be used as a quantitative proxy for growth [13]. qSIP with H_2_^18^O has been applied to terrestrial habitats [14–16], including freshwater sediments [17]. But, to our knowledge, H_2_^18^O qSIP has not yet been applied to quantify growth in estuarine sediments or under anoxic conditions.

We used H_2_^18^O qSIP [12] to quantify for the first time population-specific growth dynamics in benthic bacterial communities in response to changing redox conditions. This allowed us to test hypotheses regarding the potential physiology of several groups of uncultivated microbial groups put forth by earlier metagenomics studies, which suggested an adaptation to low oxygen and anoxic aquatic environments [6, 18]. Our results showed that hypoxia and euxinia selected for specific phylogenetic groups of uncultivated bacteria whose metabolic activity was increased, providing evidence of their optimal redox conditions for growth. Notably, establishment of benthic anoxia coincided with increased growth from numerous uncultivated groups of sulfate-reducing bacteria (SRB) that were dominated by the *Acidobacteria*, which should thus be more closely considered as an important SRB group impacting sulfur cycling in estuarine sediments. Our experimental findings validate prior hypotheses put forth by metagenomics studies indicating micro-aerophilic and anaerobic lifestyles for many groups of “microbial dark matter (MDM)”.

## Materials and methods

### Sampling

Surface sediment samples were collected in July 2016 from 1 m water depth in Sage Lot pond, a coastal lagoon connected as a sub-estuary to Waquoit Bay (Cape Cod, Massachusetts). Sage Lot pond is a small (surface area 0.17 km^2^) shallow (ca. 2 m maximum depth) lagoon surrounded by dense vegetation including salt marshes and seagrasses [19, 20]. Sage Lot pond exhibits phytoplankton chlorophyll concentration up to 90 mg L^-1^ when nitrogen inputs increase [19]. These eutrophic conditions lead to frequent benthic anoxic events [19].

### Experimental setup

We added sea salts (30 mM MgCl_2_ ٠6H_2_O, 16 mM MgSO_4_ ٠7H_2_O, 2 mM NaCO_3_, 10 mM KCl, 9 mM CaCl_2_, 450 mM NaCl) to 99% H_2_^18^O (Sigma-Aldrich, St. Louis, MO, USA) in order to create ^18^O-labeled artificial seawater (ASW). As a control, ASW was also created using diethyl pyrocarbonate (DEPC)-treated (sterile, nuclease free) water. Both waters were filter sterilized (0.2 μm). One milliliter of either ^18^O-labeled or -unlabeled (control) ASW was added to 2 g of wet surface sediment from Sage Lot Pond in 20 mL sterile glass vials containing sterile oxygen sensor spots (PreSens Precision Sensing). The oxygen sensor spot was positioned at the sediment-seawater interface to measure benthic O_2_ concentrations, and additional sensor spots were placed in the headspace of two flasks to measure gaseous O_2_ levels throughout the incubation. Incubations were set up in biological triplicate for each timepoint (7 day and 28 day). The water content of the sediments was 15% (±1%), and thus the final concentration of H_2_^18^O in the H_2_^18^O incubations was roughly 66%. After addition of labeled and unlabeled ASW, flasks were crimp sealed with gas tight gray butyl rubber stoppers. All flasks contained ca. 15 cm of oxygenated headspace and were incubated in the dark for 7 and 28 days at 8 °C. Dissolved oxygen was measured noninvasively using a fiber optic oxygen sensor (PreSens, Regensburg Germany) ca. 0.5 cm above the sediment-water interface as described previously [21]. Oxygen measurements were also performed on autoclaved sediments as a killed control. DNA from the samples was extracted and quantified from the replicate incubations at the beginning (T_0_), 7 days, and 28-day timepoints as described previously [22].

### Density gradient centrifugation and gradient fraction

DNA samples were prepared for density gradient centrifugation according to previously defined protocol for qSIP [23]. In brief, density gradient centrifugations were carried out in a TLN-100 Optima MAX-TL ultracentrifuge (Beckman Coulter, Brea, CA, USA) near-vertical rotor at 18 °C for 72 h at 165,000 × *g*. In all, 50 µl of DNA spanning from 0.5 to 1.5 µg [24] was added to a solution of cesium chloride (CsCl) and gradient buffer (0.1 M Tris, 0.1 M KCl and 1 mM EDTA) in order to achieve a starting density of 1.70 g mL^-1^ in a 3.3-mL polyallomer OptiSeal tubes (Beckman Coulter, Brea, CA, USA). After ultracentrifugation, the density gradients were fractionated into 15 equal fractions of 200 µl from the bottom of polyallomer OptiSeal tubes by using a syringe pump and fraction recovery system (Beckman Coulter, Brea, CA, USA). The density of these fractions was measured with an AR200 digital refractometer (Reichert Analytical Instruments, Depew, NY, USA). DNA was precipitated from the fractions using two volumes of polyethylene glycol with 2 µl (10 mg mL^-1^) glycogen and precipitated overnight at room temperature. DNA was pelleted by centrifugation (13,000 × *g*; 40 min), washed with 70% ethanol, and resuspended with 30 µl molecular-grade (DEPC-treated) water. DNA was quantified fluorometrically using a Qubit 4 fluorometer (Thermo Scientific).

### qPCR, 16S rRNA gene, and dsrB gene sequencing

Universal primers targeting the V4 hypervariable region of 16S ribosomal RNA (rRNA) genes were used in qPCR to determine density shifts of key genes (16S and dsrB) for each incubation. We used a version of the 16S rRNA gene 515F primer with a single-base change (in bold) to increase the coverage of archaea (515F-Y, 5′-GTG**Y**CAGCMGCCGCGGTAA-3′; [25]). All qPCR reactions were carried out as described previously using the Eppendorf EpMotion 5070 pipetting robot that has <5% technical variation [23]. Each density fraction was also screened using qPCR for SRB with primer pairs targeting the dissimilatory sulfite reductase β-subunit genes (dsrB) according to a previously published assay [26–28]. We chose to focus on the *dsrB* gene because a large database exists for *dsrB* sequences recovered from environmental samples that we could compare our data against [29] (www.microbial-ecology.net/download). qPCR standards consisted of 10-fold dilution series of the genes of interest that were PCR amplified from the sample at 40 cycles using the same primers. Prior to creating the dilution series, the correct size of amplified standard was confirmed via gel electrophoresis, gel extracted, and quantified with a Qubit. Reaction efficiencies in all qPCR assays were between 90 and 110% with *r*^2 ^> 0.98 for the standards. *dsrB* amplicons were cloned and sequenced via sanger sequencing from density fractions at the 28-day incubation timepoint that exhibited ^18^O labeling, in the density range 1.70–1.71 g mL^-1^.

Two 16S PCR amplicons from each density fraction (technical replicates to reduce PCR bias) were pooled and sequenced on the Illumina MiniSeq as described previously [30]. To account for the influence of contamination, we included barcoded aerosol (laboratory dust) and kit reagents (DNA extraction blanks) samples.

*dsrB* amplicons were gel extracted and cloned using the TOPO TA cloning kit (Invitrogen, Life Sciences) according to the manufacturer instructions. A total of 132 clones were picked, the insert size confirmed via PCR, and those clones having the correct *dsrB* size (89 clones) were Sanger sequenced bidirectionally. The forward and reverse Sanger reads were used to create *dsrB* contig sequences in CodonCode Aligner version 8.0.2 (CodonCode Corporation, MA, USA).

### Bioinformatic analysis

The Illumina reads were quality trimmed and assembled using USEARCH version 10.0.240 with the default parameters [31] resulting in 6.8 million quality checked V4 reads. Reads were then de novo clustered at 97% identity using UPARSE; OTUs represented by a single sequence were discarded [32]. Taxonomic assignments were generated by QIIME 1.9.1 [33] using the implemented BLAST method against the SILVA rRNA gene database release 132 [34]. After that, only operational taxonomic units (OTUs) >12 sequences in total in each replicate for the control and SIP-labeled fractions were selected for further study [23, 35]. OTUs detected in the contaminant datasets were removed from all downstream analysis if the total number of sequences in the contaminant sample was greater than the experimental sample. Working with this “cleaned” dataset, 598 OTUs and 523 OTUs from 7 days and 28 days incubations were used for downstream analyses.

Observed excess atom ^18^O fractions (EAFs) were calculated for each taxon as described previously [13] using a qSIP workflow embedded in the HTS-SIP R package [36]. To calculate the bootstrap confidence intervals (CI) for significant isotopic incorporation, bootstrap replicates (*n* = 1000) were run with the HTS-SIP R package [36]; an OTU was considered as having isotopic incorporation (true positive) if the lower CI was >0 [13].

Phylogenetic analyses were performed in SeaView [37] following alignment with MUSCLE [38]. Maximum likelihood (ML) with selected substitution model as general time reversable (GTR) was performed with PhyML version 3.0 [39]. *dsrB* gene translation was performed using EMBOSS Transeq [40]. W-IQ-TREE (http://iqtree.cibiv.univie.ac.at) was used to find the best model using Model Finder [41, 42], which resulted in LG + G4 model. Trees were visualized and edited using iTOL [43]. Statistical analyses and plots were performed using R.Studio Version 3.3.0 [44]. Blomberg’s K [45] and Pagel’s λ [46] tests for significantly non-random phylogenetic distributions of growing patterns from qSIP were calculated on all OTUs (labeled and unlabeled) using the phylosignal R package [47]. Both indices test species’ traits under a Brownian motion model (BM) of trait evolution; that is whether or not the distribution of traits across different phylogenetic groups is random or non-random. The BM assigns a 0 value to indicate phylogenetic independence (random phylogenetic distribution of traits) and values close to 1 for a strong phylogenetic signal (non-random phylogenetic distribution of traits) [48–50].

The sizes of the growing and dying fractions of each population, and their rates of change, was calculated using a model developed to determine population growth and mortality rates from ^18^O-qSIP data [51]. For all calculations, bootstrap resampling of replicates within each treatment was used to reproduce the uncertainty and 90% CIs were estimated. These calculations were performed in R using the code at https://bitbucket.org/QuantitativeSIP/qsip_repo [51]. Sequence data were entered in the NCBI Short Read Archive under BioProject ID PRJNA498588.

## Results

### Dissolved oxygen measurements

**Fig. 1:**
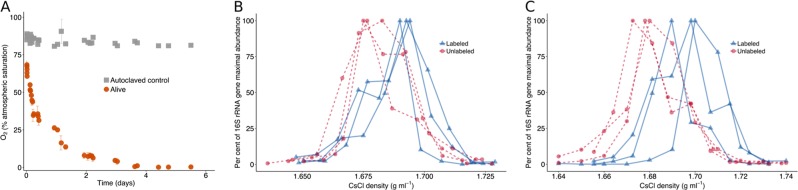
**a** Benthic O_2_ concentrations during the incubation and killed (autoclaved) control. **b**, **c** Quantification of 16S rRNA gene copies across CsCl density gradient fractions after 7 (**b**) and 28 days (**c**). ^18^O water replicates are represented by blue solid lines with triangles and unlabeled replicates (control) are represented by red dashed lines with circles. The *y* axis represents the relative abundance of 16S rRNA genes quantified with qPCR, normalized to maximal abundance across all density fractions

The sediment-water interface was well-oxygenated at the beginning of the incubation (70% atm. saturation), which declined exponentially during the first week until reaching 0% atm. saturation after day 5 (Fig. 1a). This was not observed in the killed control, showing that the rapid drawdown of benthic O_2_ was due to respiration. Small fluctuations in the oxygen measurements in the killed control were likely due to temperature fluctuations of the incubator itself (±1 °C), since the non-invasive fiber optic oxygen sensor spots are temperature sensitive [21]. By the end of the experiment (28 days), the sediments had turned from a gray-brown color to black, indicating the presence of iron–sulfur minerals (e.g., FeS and FeS_2_), and upon opening the vials sulfide could be smelled. Thus, while the headspace contained oxygen at the beginning of the experiment, the sediments had become anoxic and sulfidic (euxinic) by the end of the incubation period.

### qSIP of 16S rRNA genes

^18^O labeling of 16S rRNA genes was observed at 7 and 28 days, with 16S rRNA genes at 28 days exhibiting a higher degree of labeling compared with 7 days (Figs. 1b, c). The decrease in oxygen (Fig. 1a) was mirrored by a decrease in 16S rRNA gene copies: the total number of 16S rRNA gene copies per gram wet sediment decreased during the first week, from 2.5 ( ± 0.12) × 10^8^ at T_0_ to 1.4 ( ± 0.09) × 10^8^ at 7 days, and then decreased further after 28 days to 1.1 ( ± 0.04) × 10^8^. This indicated net microbial death with a fraction of the community maintaining growth during the incubation.

**Fig. 2:**
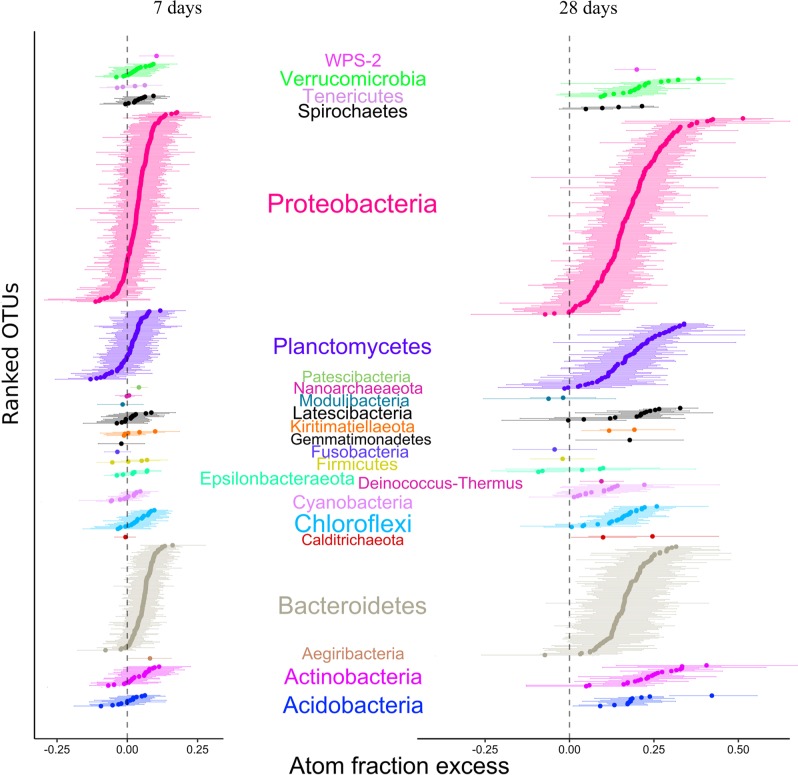
OTU-specific shifts in the median atom fraction excess (^18^O) of OTUs with 90% confidence interval (CI). OTUs were colored by phylum. OTUs that do not have a 90% CI overlapping with 0 are considered to be ^18^O labeled

The composition of microbial populations at 7 and 28 days were markedly different (analysis of variance; *F* = 3991, *p* < 0.001), but dominated by the same phyla in nearly equal proportion (Figure S1). In total, 443 OTUs were detected at both timepoints, whereas 235 OTUs were detected at only a single timepoint (Figure S1). In all, 128 OTUs were ^18^O labeled after 7 days, which increased to 395 OTUs after 28 days (Figure S1). Of the ^18^O-labeled OTUs at day 7, *Bacteroidetes* were the most abundant taxa with 67 OTUs (49.4% of the ^18^O-labeled OTUs), followed by 39 OTUs affiliated with *Proteobacteria* (42.1% of the ^18^O-labeled OTUs) (Fig. 2 and S1). On the other hand, the 395 ^18^O-labeled OTUs at day 28 were comprised mostly of *Proteobacteria* (157 OTUs, 61.4% of ^18^O-labeled OTUs), followed by *Bacteroidetes* (94 OTUs; 18.1% of ^18^O-labeled OTUs) and *Planctomycetes* (53 OTUs; 8.71% of ^18^O-labeled OTUs) (Fig. 2 and S1).

**Fig. 3:**
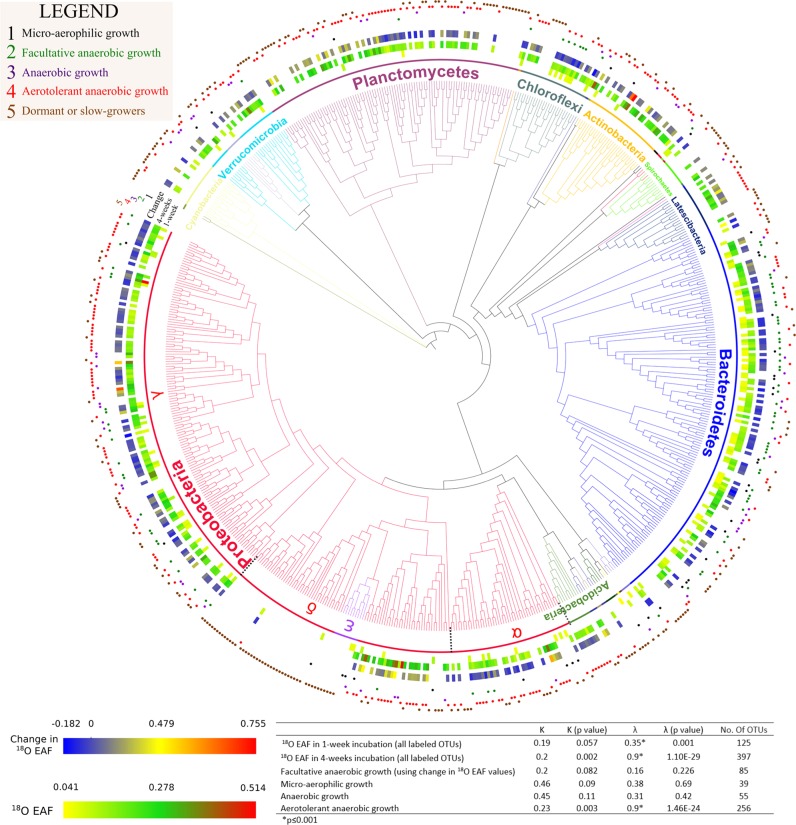
Phylogeny of bacterial taxa detected and their extent of ^18^O labeling at 7 and 28 days. The inner circles correspond to excess atom fraction (EAF) ^18^O values of labeled taxa after 7 and 28 days of incubation. The outer ring of the heatmap represents the EAF change between the timepoints. The numbered and colored circles represent the growth mode of the OTUs and the legend defines the growth mode of categories explained in result section. Bottom panel shows phylogenetic signal tests (Blomberg’s K and Pagel’s λ) and corresponding p-values for labeled taxa and growth mode categories

Although the scope of this study is to determine the growing microorganisms, non-growing cells due to dormancy and or slow growing cells are represented in our results as those OTUs that did not become labeled. Accordingly, a total of 248 OTUs constituted non-growing microbes (unlabeled) in both incubations (Fig. 3). The non-growing or slow growing bacterial groups were dominated by OTUs affiliated with sulfate-reducing lineages in the *Deltaproteobacteria* (42 OTUs), *Planctomycetes* (38 OTUs), *Epsilonbacteraeota* (7 OTUs), and *Spirochaetes* (9 OTUs) (Fig. 3).

Growing bacterial OTUs affiliated with MDM [18] candidate phyla *Aegiribacteria* (1 OTU, 0.08 EAF) and *Patescibacteria* (1 OTU, 0.04 EAF) were detected only in the 7-day incubations (Fig. 2 and Table 1). Labeled OTUs affiliated with MDM groups after 28 days included *Latescibacteria* (10 OTUs, 0.24 ± 0.04 EAF) and *Calditrichaeota* (1 OTU, 0.24 EAF). The only MDM group that was ^18^O labeled at both 7- and 28-day timepoints was the candidate phylum *WPS-2* (Fig. 2 and Table 1).

**Table 1:** A summary of growth by uncultivated taxa, previously designated as “microbial dark matter”, under various redox conditions

Group	Growth mode	EAF values (number of OTUs)	*r*-Value (net population growth rate)	*b-*Value (rates of reproduction)	*d*-Value^a^ (mortality rate)
Aegiribacteria	Micro-aerophilic	0.08 (*n* = 1)	-0.202	0.081	-0.288
Latescibacteria	Aerotolerant anaerobic	0.013 ± 0.008 (*n* = 10)	-0.227	-0.038	-0.191
Gracilibacteria	Micro-aerophilic	0.04 (*n* = 1)^b^	-0.172	-0.041	-0.136
Calditrichaceae	Aerotolerant anaerobic	-0.007 (*n* = 1)	-0.214	-0.018	-0.197
WPS-2	Facultative anaerobic	0.1 (*n* = 1)^b^	-0.339	0.079	-0.421
28 days of incubation					
Gracilibacteria	Aerotolerant anaerobic	0.17 ± 0.073 (*n* = 2)^b^	-0.136	0.023	-0.171
Latescibacteria	Aerotolerant anaerobic	0.19 ± 0.023 (*n* = 14)^c^	-0.373	0.019	-0.392
WPS-2	Facultative anaerobic	0.2 (*n* = 1)^b^	-0.422	0.014	-0.436

^a^*r*, *b*, and *d* values correspond to the rates per day (d^-1^)

^b^Significantly growing microorganisms based on qSIP (i.e., lower boundary of bootstrap is >0)

^c^Most of the Latescibacteria grew (10 OTUs out of 14)

The change in oxygen concentrations over the course of the experiment allowed us to group OTU growth into five categories based on the pattern of ^18^O labeling at 7 days (micro-oxic conditions) and 28 days (anoxic conditions) (Fig. 3) [1]. Micro-aerophilic growth was defined as ^18^O-labeled OTUs detected only at day 7, and not at 28 days [2]. Anaerobic growth was defined as ^18^O-labeled OTUs detected only after establishment of euxinic conditions at day 28, and not at 7 days [3]. Facultative anaerobic growth was defined as ^18^O-labeled OTUs overlapping between both timepoints [4]. Aerotolerant anaerobic growth was defined as OTUs detected at both timepoints, but only ^18^O labeled during anoxic and sulfidic conditions [5]. Dormant or slow growing microorganisms were defined as OTUs that were not ^18^O labeled at either timepoint.

### ^18^O labeling of dsrB genes

**Fig. 4:**
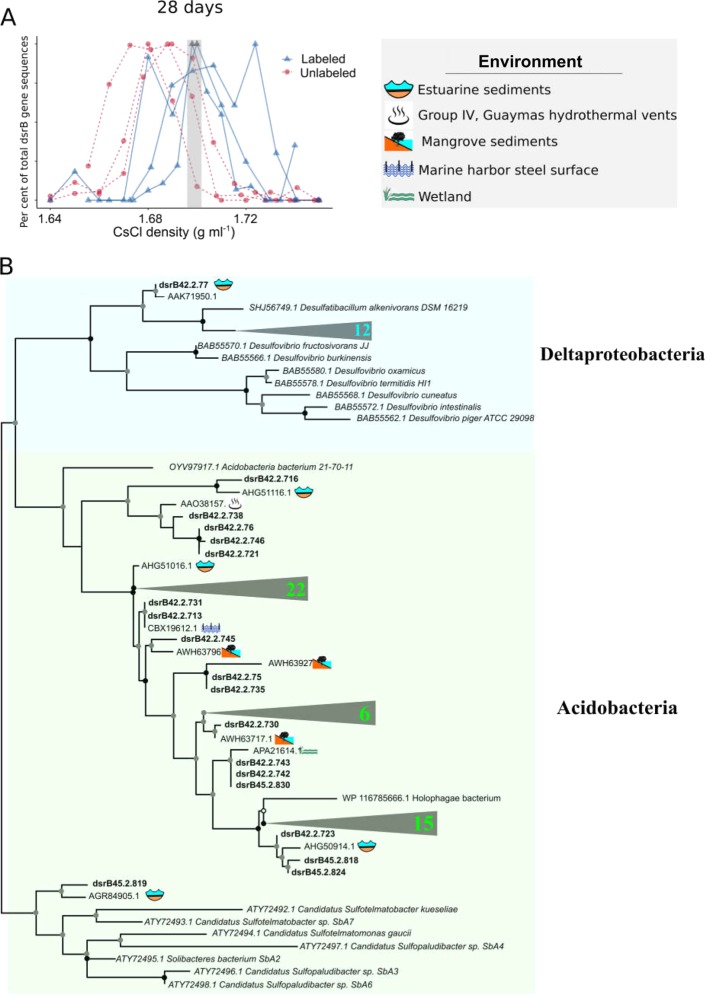
**a** Quantification of *dsrB* gene copies across CsCl density gradient fractions after 28 days. ^18^O water replicates are represented by blue solid lines with triangles and unlabeled replicates (control) are represented by red dashed lines with circles. The y axis represents the relative abundance of *dsrB* genes quantified with qPCR normalized to maximal abundance across all density fractions. **b** Phylogenetic tree of ^18^O-labeled *dsrB* genes including their most similar sequences from the NCBI nr database, bold sequences indicate those from this study. Collapsed clades (triangles) show the number of ^18^O-labeled *dsrB* gene sequences contained within the clade. Black circles at nodes represent bootstrap support of 90%, gray circles represent bootstrap support from 70 to 90%, and white circles represent bootstrap support from 70 to 50%

Bacterial *dsrB* genes exhibited ^18^O labeling only after development of euxinic conditions sampled at 28 days of incubation, with peak DNA buoyant density (BD) of 1.71 (±0.008) g mL^-1^, which was greater than the control where the peak was 1.686 (±0.003) (Fig. 4a). This corresponds to an increase in the atomic enrichment percentage of >20%, which is typically regarded as the threshold for significant isotopic labeling [52]. In total, 89 *dsrB* sequences were obtained by molecular cloning from the density fractions that exhibited peak ^18^O labeling (Fig. 4a). Most of the ^18^O-labeled *dsrB* sequences were affiliated with novel groups of uncultivated *Acidobacteria* SRB (61 sequences, 82% of total), whereas the remainder of ^18^O-labeled *dsrB* sequences (13 sequences, 18% of total) were related to Deltaproteobacteria SRB (Fig. 4b). The ^18^O-labeled *Acidobacteria dsrB* genes include a clade of four *dsrB* sequences with close relation to a novel clade of SRB originally described as “novel *dsrB* Group IV” from the Guaymas hydrothermal vent [53], raising the possibility that this group of *Acidobacteria* contains both thermophilic and mesophilic SRB.

### Growth and death dynamics of ^18^O-labeled populations

**Fig. 5:**
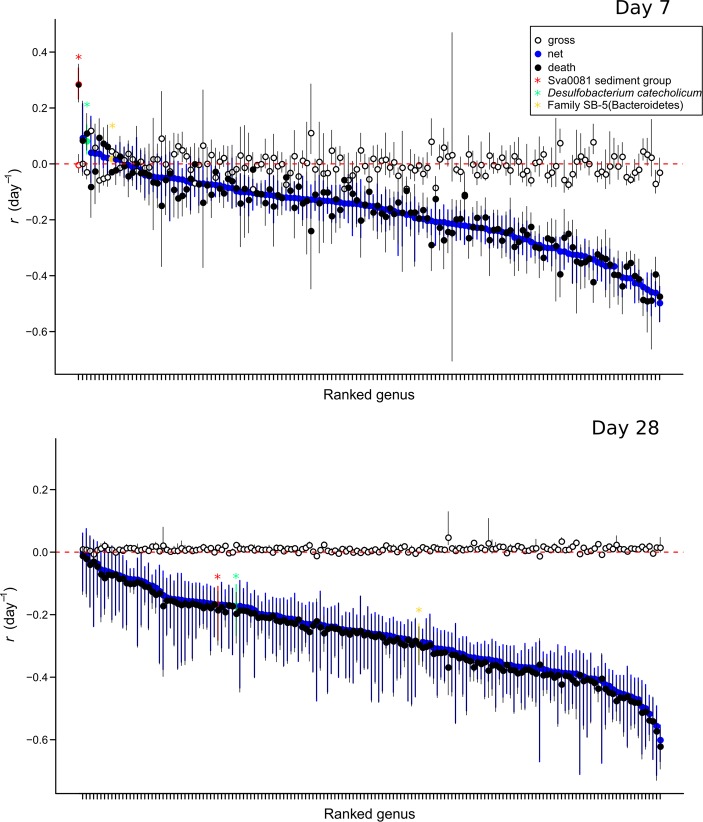
Population growth rates (r) at 7 and 28 day timepoints. After 28 days, all taxa exhibited mortality rates greater than reproduction rates. Points indicate bootstrapped medians and bars show 90% confidence intervals for each OTU. The positive net production rates here are considered as statistically significant increase in the populations if the bootstraps are not crossing the zero. Open circles: rates of reproduction, black filled circles: mortality rates, blue filled circles: net reproduction rates. The only groups that exhibiting significant net production at day 7 are indicated with asterisks (see legend)

After 7 days, most genera exhibited gross reproduction and three OTUs exhibited significant net production (defined as 90% CI in the growth/death model not overlapping 0), affiliated with Sva0081 sediment group (*Desulfobacteraceae*), *Desulfobacterium catecholicum*, and SB-5 family of *Bacteroidetes* (Fig. 5). The rate of mortality per genus was higher at day 28 than day 7, indicating that establishment of euxinic conditions caused the majority of cells per genus to die faster than they grew (Fig. 5). However, despite the higher net mortality rates, many exhibited relatively low positive gross reproduction rates (Fig. 5) indicating that a smaller proportion of individuals per population were actively growing. This is consistent with the result that most of the OTUs were ^18^O labeled at this timepoint. We also calculated the whole-assemblage turnover estimated via qSIP using the developed model of Koch et al. [51]. The seven-day incubation had an average community turnover value of 0.28 d^-1^ (90% CI: 0.219–0.36 d^-1^), whereas 28 days of incubation had an average community turnover value of 0.371 d^-1^ (90% CI: 0.315–0.476 d^-1^).

## Discussion

Dissolved oxygen has declined in ocean water in the past five decades due to the increase in global temperature [54], resulting in the expansion of oxygen minimum zones (OMZs) in the oceans [55]. In coastal settings, increased human activity such as high fertilizer use has caused widespread eutrophication and recurrent bottom water anoxia that influences the benthos [19, 56, 57]. The effects of such benthic anoxia on the growth of diverse bacterial populations driving elemental cycles is poorly understood. We used ^18^O-qSIP to quantify for the first time to quantify the growing bacterial populations in response to benthic oxygen depletion, including many groups for which there currently exists no cultured representatives.

### Assessing effects of incubation conditions

Although the sediment surface became oxygen depleted after 5 days of incubation, there was an oxygenated headspace, and O_2_ measurements of the headspace confirmed oxygen throughout the 28-day incubations at concentrations of 90–80% atmospheric saturation (data not shown). The gradual depletion of oxygen at the benthic interface during the first week thus indicates a shoaling of the oxic–anoxic transition zone within the sediments into the overlying ASW. Benthic anoxia is a common feature of the sampled environment in Waquoit Bay (Cape Cod, USA), where increased nitrogen input to the watershed through atmospheric deposition, fertilizer, and wastewater has led to an increase in primary productivity and recurrent bottom water anoxia [19, 56]. This phenomenon is also known to occur in the marine environment, for example, in the Benguela upwelling system where summer time water column stratification results in a shoaling of the sediment oxic–anoxic transition zone several meters upwards into the bottom waters and where sulfide accumulates to high levels on the continental shelf [58]. Thus, our experimental conditions are relevant to naturally occurring benthic habitats in estuaries and continental shelf settings that are especially prone to the development of anoxia. Dormancy by anaerobic bacteria during periods of oxygenation at the sediment surface may explain how they survive until favorable anoxic conditions are re-established, for example, after burial deep below the subseafloor in anoxic sediments [59].

Given the detection limit of the fiber optic oxygen measurements (ca. 0.5 % atmospheric saturation), we cannot rule out the presence of trace amounts of dissolved oxygen within the overlying seawater or sediment. Nevertheless, the appearance of black color throughout the sediments and the strong smell of sulfide at the end of the 28-day incubation strongly indicated anoxic conditions in the sediments by the end of the experiment. The strong smell of sulfide suggests that the black color reflected formation of amorphous iron–sulfur compounds, caused by a reaction between oxidized Fe(III) with HS^-^ [60]. As we sampled the entire sediment slurry for our qSIP analysis, we likely sampled a mixture of both anoxic and micro-aerophilic habitats that were present as a steep redox gradient within the flasks. Therefore, the active microbes detected at 7 days probably represent micro-aerophilic bacteria, whereas active microbes sampled at 28 days were living under predominantly anoxic and sulfidic conditions.

It is likely that anoxic microsites likely developed within the sediment in the first 7 days, potentially complicating the categorization of aerobic, facultatively anaerobic, and aerotolerant growth in OTUs detected at this timepoint. Anaerobic SRB can remain active in oxidized marine sediments within anoxic microniches [61], but some SRB are aerotolerant since they can grow (albeit slightly) in the presence of oxygen [reviewed in [62]]. The total area occupied by anoxic microsites should be much smaller compared with the rest of the bulk sediment sampled that experienced oxygen during the first 7 days. Thus, the dominant OTUs detected at day 7 are unlikely to be those living in anoxic microsites. But, it is possible that some strictly anaerobic populations survived at low abundance under oxygen exposure during the first 7 days within anoxic microsites.

It is also likely that after the 28 days of incubation the label turned over, and a second generation of microbial cells became labeled not only from ^18^O water but also from ^18^O-labeled organic compounds that formed earlier. In this case, some of the labeling detected at the 28-day timepoint could have been due to assimilation of ^18^O-labeled organic compounds. However, the concentration of any ^18^O transferred to organic matter would be diluted within the much larger (unlabeled) dissolved organic matter pool. This would then be further diluted several orders of magnitude by the ^18^O label in the water that was present at a molar concentration. Thus, the potential assimilation of ^18^O-labeled organic compounds is unlikely to affect the conclusion that the degree of ^18^O labeling is a measure of assimilation of ^18^O from water, and thus activity, in growing populations.

### Phylogenetic grouping of redox-specific activities

The oxygenated concentrations during the first week and development of anoxic and sulfidic conditions at 28 days allowed us classify ^18^O-labeled OTUs detected between these two timepoints into four categories of growth (Fig. 3) [1]; *micro-aerophilic growth* [2], *facultative anaerobic growth* [3], *anaerobic growth*, and [4] *aerotolerant anaerobic growth* (see Results for category definitions). The non-growing microorganisms were also considered as an additional category [5]: *dormant* or *slow-growers*. We recognize that because oxygen was present in the headspace throughout the incubation, strict anaerobic growth cannot be unequivocally assigned to the OTUs. But, given the increase in their growth later in the incubation after the onset of euxinic conditions at the sediment-water interface were established, we interpret this as an indicator of anaerobic growth. We also recognize that our designation of facultative versus aerotolerant anaerobic growth is arbitrary, but is used here to differentiate between those OTUs labeled at day 28 present also at day 7 that were, or were not, ^18^O labeled at day 7.

OTUs exhibiting micro-aerophilic growth had a relatively weak phylogenetic distribution across the phylogeny (λ = 0.38) (Fig. 3). In contrast, ^18^O-labeled OTUs exhibiting anaerobic growth corresponded to non-random phylogenetic groupings (λ = 0.9 and 0.87, respectively), implying that traits conferring anaerobic growth were conserved in the sampled communities. ^18^O-labeled organisms detected at both timepoints (facultative anaerobes) did not display a significant phylogenetic pattern (λ = 0.16), suggesting that facultative anaerobic growth was not a conserved trait in our sampled communities.

### Populations exhibiting micro-aerophilic growth

A total of 47 OTUs affiliated with orders *Flavobacteriales* and *Chitinophagales* within the phylum *Bacteroidetes* were the most active growing bacteria after 7 days, maintaining metabolic activity under suboxic conditions. Similarly, seven OTUs affiliated with order *Anaerolineae* (*Chloroflexi*) were ^18^O labeled at day 7 (0.08 ± 0.008 EAF). Furthermore, 26 OTUs affiliated with *Gammaproteobacteria* (mainly orders *Cellvibrionales* and *Thiotrichales*) were labeled at day 7, and demonstrated relatively high EAF values (0.102 ± 0.031) indicating micro-aerophilic growth (Fig. 3).

^18^O-labeled OTUs affiliated with candidate class *Gracilibacteria* were observed solely at day 7, consistent with a micro-aerophilic growth (Table 1). *Gracilibacteria* have a cytochrome/quinol oxidase [18], most specifically cytochrome *bd*, which is implicated in ameliorating oxidative stress effects [reviewed in [63]]. Cytochrome *bd* could thus help to explain the higher growth of *Gracilibacteria* under low oxygen conditions.

### Populations exhibiting anaerobic growth

There were 256 OTUs that exhibited a pattern of ^18^O labeling consistent with aerotolerant anaerobic growth (Fig. 3). They are apparently capable of surviving in the presence of oxygen during the beginning of the incubation, but their growth was maximized under anoxic conditions. The majority of these OTUs were affiliated with the *Gammaproteobacteria* (69 OTUs; 0.18 ± 0.06 EAF) and *Deltaproteobacteria* (19 OTUs; 0.29 ± 0.02 EAF). The highest ^18^O-labeled OTU was affiliated with genus *Zhongshania* that contains the facultative anaerobic heterotrophic species *Zhongshania aliphaticivorans* SM-2^T^ [64]. In addition, one of the ^18^O-labeled *Gammaproteobacteria* OTUs was affiliated with the JTB255/Woesiaceae clade, which have been identified as the most dominant dark carbon-fixing microbes with a capacity to oxidize reduced sulfur compounds in anoxic and suboxic coastal sediments [65]. The genus *Sandaracinus* in *Deltaproteobacteria* known to degrade complex polysaccharides [66], also exhibited aerotolerant anaerobic growth. At day 28 after the onset of euxinic conditions, 10 OTUs affiliated with the *Latescibacteria* were detected that grew anaerobically (Table 1). This is consistent with their proposed fermentative mode of metabolism in anoxic sediment and water columns [18, 67].

OTUs affiliated with the known SRB genera [68] *Desulforhopalus*, *Desulfosarcina*, *Desulfobulbus, Desulfopila, Desulfobacter, Desulfotignum*, and *Desulfatitalea* were well-represented comprising 9% (42 OTUs) and 8% (26 OTUs) of the total sequences at days 7 and 28, respectively. However, only one SRB OTU was ^18^O labeled, which occurred at day 7 and was affiliated with the SRB genus *Desulfobulbus* (0.10 EAF). The relatively small number of growing Deltaproteobacteria SRB is low compared with the more numerous populations of sulfate-reducing *Acidobacteria* that have higher ^18^O EAF values (Figs. 3, 4). This indicates that these anaerobic *Acidobacteria* SRB were growing faster compared with the Deltaproteobacteria SRB.

*Acidobacteria* belong to several newly discovered groups of SRB, showing that dissimilatory sulfur metabolism is more widespread than previously thought [29, 69]. For example, *Acidobacteria* with a dissimilatory sulfur metabolism have been recently identified in acidic peatland [69, 70] and a DNA-SIP study [71] showed activity of *dsrAB*-containing organisms derived from *Acidobacteria* [70]. Our study shows that in addition to acidic peatland, *dsrB* carrying *Acidobacteria* grow in anoxic estuarine sediments with a relatively fast rate. Their increased growth rate and activity compared to Deltaproteobacteria SRB shown here implies that they should have a large impact on dissimilatory sulfur cycling under anoxic conditions.

Our finding that the majority of deltaproteobacterial sulfate reducers had minimal growth is in line with the previous reports that their mean in situ doubling times are on the order of months to years [72]. Metabolic activity (e.g., rRNA synthesis) in natural microbial communities is typically followed by cell division [16], but metabolic activity of non-growing organisms can also influence biogeochemical cycles [72]. For example, the increase in activity of SRB Candidatus *Desulfosporosinus infrequens* can occur independent of cell growth-associated processes [73]. Thus, the low degree of ^18^O labeling in abundant deltaproteobacteria SRB seen in our study (Fig. 3) may relate to a different ecophysiological strategy (e.g., slow growth) compared with the faster growing *Acidobacteria* SRB.

Within the Planctomycetes, members of the uncultured OM190 clade were abundant (44% total Planctomycetes) and only became ^18^O labeled at day 28 (Fig. 3), indicating anaerobic growth. Representatives of the OM190 clade closely related to anaerobic ammonia oxidizing bacteria (anammox) were also detected in hypoxic estuarine surface sediments in the East China Sea [74]. The anaerobic growth of OM190 clade organisms seen here is consistent with a potential anammox metabolism.

The ^18^O-labeled Actinobacteria were dominated by OTUs most closely related to the enigmatic Sva0996 actinobacterial clade first described from marine sediments [75], the “*Candidatus* Actinomarinales” [76], and *Rhodococcus*. While aerobic growth of *Rhodococcus* is well known as it relates to hydrocarbon degradation [77], our results showing facultative anaerobic growth indicate that *Rhodococcus* has potential to degrade hydrocarbons also under anoxic conditions in sediments. A facultative anaerobic lifestyle also explains why *Rhodococcus* are often found in deep subseafloor anoxic marine sediments [78].

The ^18^O-labeled Verrucomicrobia were dominated by OTUs affiliated with the uncultured DEV007 clade, first reported from the Elbe River in Germany (unpublished data, accession number: AJ401107). ^18^O-labeled OTUs affiliated with DEV007 were labeled at 7 days and became increasingly labeled after 28 days and the establishment of anoxic conditions (Fig. 2). This is consistent with biogeographic surveys that have detected this group in anoxic estuarine sediments (unpublished data, accession number: JN672646), OMZs (unpublished data, accession number: MG875625), and marine sediments [79]. However, the DEV007 clade is also found in oxic seawater attached to particles [35]. Our ^18^O labeling results showing a facultative anaerobic growth of the DEV007 clade explains this wide biogeographic range.

An OTU affiliated with the WPS-2 (Writtenberg Polluted Soil) clade was the only MDM group exhibiting facultative anaerobic growth (Fig. 2). The WPS clade was first described in a study of polychlorinated biphenyl-polluted soil in Germany [80] and was since detected in a wide range of oxic and anoxic environments [81]. The facultatively anaerobic growth shown here could potentially explain the ability of the WPS-2 group to survive in a large number of habitats with widely varying redox states.

### Growth and death dynamics of ^18^O-labeled populations

Although total microbial abundance decreased over the incubation by nearly an order of magnitude, 16S rRNA genes became increasingly enriched in ^18^O (Fig. 1c). This raised the possibility that within populations, a high number of cells were dying while a smaller number were growing. In order to investigate this possibility further, we applied a model [51] that uses the ^18^O-qSIP data to calculate the number of ^18^O-labeled and -unlabeled 16S rRNA genes per OTU and the changes in their ratio over time to estimate rates of gross reproduction, mortality, and net production of individual OTUs (Fig. 5).

After seven days, only three OTUs exhibited net production (defined as net production 90% CI not overlapping zero), which were affiliated with the Sva0081 sediment group (*Desulfobacteraceae*), *Desulfobacterium catecholicum*, and SB-5 family of *Bacteroidetes*. Our analysis suggests that the Sva0081 marine benthic group (MBG), a putative group of SRB that are an important sink of acetate [82] and H_2_ [83] in coastal marine sediments, was one of the fastest growing populations under micro-aerophilic conditions at day seven (Fig. 5). The increased micro-aerophilic growth of Sva0081-MBG individuals is consistent with their higher acetate uptake rates in oxic–suboxic surface layer sediment slurries [82]. As Sva0081-MBG are H_2_ oxidizers [83], it is possible that the fast growing Sva0081-MBG individuals at the oxic–anoxic interface were also utilizing fermentation-derived H_2_ diffusing upwards from the deeper anoxic sediments. *Desulfobacterium catecholicum*, which exhibited net production at day seven (Fig. 5), is a mesophilic SRB that can also perform dissimilatory reduction of nitrate to ammonium [84]. This would explain its higher growth rate under the micro-aerophilic conditions at day seven during which nitrate was possibly still present. The SB-5 group of Bacteroidetes belongs to the Cytophagales and was previously detected in a benzene degrading, sulfate-reducing consortium [85]. The high net production under micro-aerophilic conditions indicates this group was growing faster compared with most other OTUs (Fig. 5).

After 28 days, most of the OTUs were ^18^O labeled (Fig 2). However, the rate of mortality per genus was higher at day 28 than day 7, indicating that establishment of euxinic conditions caused the majority of cells per genus to die faster than they grew (Fig. 5). However, despite the higher the net mortality rates, many exhibited relatively low positive gross reproduction rates. This indicates that a smaller proportion of individuals within each OTU were actively growing, while the majority of individuals were dying. This small fraction of active cells per population explains how nearly all OTUs became ^18^O labeled by day 28, despite of the increase in total microbial mortality.

Larger mortality compared with production at the end of the 28-day incubation could be partly explained by a decrease in electron donors (e.g., organic matter) and acceptors (e.g., nitrate, Fe(III), and sulfate). Indeed, under normal conditions organic matter and dissolved ions from seawater (e.g., sulfate) would be slowly but continuously entering the sediment to fuel new microbial growth. In our incubations, however, nutrients were constantly being depleted without replacement. Some of the inactive populations could also be explained by an increase in dormant cells due to unfavorable environmental conditions [9].

## Conclusions

Our findings provide the first experimental evidence demonstrating the redox conditions promoting growth in several groups of uncultured “microbial dark matter”, validating hypotheses put forth by earlier metagenomics studies. The data help to explain previously observed biogeographic patterns for many uncultivated groups of bacteria that tend to correlate with anoxic or low oxygen conditions in aquatic habitats. This information could be helpful to guide future cultivation efforts for groups of ubiquitous, yet uncultured, bacterial taxa.

## Supplementary information

Fig S1

## References

[1] Boynton WR, Kemp WM (1985). Nutrient regeneration and oxygen consumption by sediments along an estuarine salinity gradient. Mar Ecol Progress Ser Oldendorf.

[2] Nixon SW, Neilson BJ, Cronin LE (1981). Remineralization and nutrient cycling in coastal marine ecosystems. Estuaries and nutrients.

[3] Glud RN (2008). Oxygen dynamics of marine sediments. Mar Biol Res.

[4] Diaz RJ, Rosenberg R (2008). Spreading dead zones and consequences for marine ecosystems. Science.

[5] Middelburg JJ, Klaver G, Nieuwenhuize J, Markusse RM, Vlug T, van der Nat FJWA (1995). Nitrous oxide emissions from estuarine intertidal sediments. Hydrobiologia.

[6] Baker BJ, Lazar CS, Teske AP, Dick GJ (2015). Genomic resolution of linkages in carbon, nitrogen, and sulfur cycling among widespread estuary sediment bacteria. Microbiome.

[7] Luna GM, Manini E, Danovaro R (2002). Large fraction of dead and inactive bacteria in coastal marine sediments: comparison of protocols for determination and ecological significance. Appl Environ Microbiol.

[8] Bowen JL, Ward BB, Morrison HG, Hobbie JE, Valiela I, Deegan LA (2011). Microbial community composition in sediments resists perturbation by nutrient enrichment. ISME J.

[9] Kearns PJ, Angell JH, Howard EM, Deegan LA, Stanley RHR, Bowen JL (2016). Nutrient enrichment induces dormancy and decreases diversity of active bacteria in salt marsh sediments. Nat Commun.

[10] Schwartz E (2007). Characterization of growing microorganisms in soil by stable isotope probing with H218O. Appl Environ Microbiol.

[11] Blazewicz SJ, Schwartz E, Firestone MK (2014). Growth and death of bacteria and fungi underlie rainfall-induced carbon dioxide pulses from seasonally dried soil. Ecology.

[12] Schwartz E, Hayer M, Hungate BA, Koch BJ, McHugh TA, Mercurio W (2016). Stable isotope probing with (18)O-water to investigate microbial growth and death in environmental samples. Curr Opin Biotechnol.

[13] Hungate BA, Mau RL, Schwartz E, Caporaso JG, Dijkstra P, van Gestel N (2015). Quantitative microbial ecology through stable isotope probing. Appl Environ Microbiol.

[14] Woods A, Watwood M, Schwartz E (2011). Identification of a toluene-degrading bacterium from a soil sample through H218O DNA stable isotope probing. Appl Environ Microbiol.

[15] Schwartz E, Van Horn DJ, Buelow HN, Okie JG, Gooseff MN, Barrett JE (2014). Characterization of growing bacterial populations in McMurdo Dry Valley soils through stable isotope probing with 18O-water. FEMS Microbiol Ecol.

[16] Papp Katerina, Hungate Bruce A., Schwartz Egbert (2018). Microbial rRNA Synthesis and Growth Compared through Quantitative Stable Isotope Probing with H218O. Applied and Environmental Microbiology.

[17] Hayer M, Schwartz E, Marks JC, Koch BJ, Morrissey EM, Schuettenberg AA (2016). Identification of growing bacteria during litter decomposition in freshwater through H218O quantitative stable isotope probing. Environ Microbiol Rep.

[18] Rinke C, Schwientek P, Sczyrba A, Ivanova NN, Anderson IJ, Cheng JF (2013). Insights into the phylogeny and coding potential of microbial dark matter. Nature.

[19] Valiela I, Foreman K, LaMontagne M, Hersh D, Costa J, Peckol P (1992). Couplings of watersheds and coastal waters: sources and consequences of nutrient enrichment in Waquoit Bay, Massachusetts. Estuaries.

[20] Wang ZA, Kroeger KD, Ganju NK, Gonneea ME, Chu SN (2016). Intertidal salt marshes as an important source of inorganic carbon to the coastal ocean. Limnol Oceanogr.

[21] Ortega-Arbulu AS, Pichler M, Vuillemin A, Orsi WD. Effects of organic matter and low oxygen on the mycobenthos in a coastal lagoon. Environ Microbiol. 2019;21:374–88. 10.1111/1462-2920.14469 PMC7379666 30411473

[22] Orsi William D., Wilken Susanne, del Campo Javier, Heger Thierry, James Erick, Richards Thomas A., Keeling Patrick J., Worden Alexandra Z., Santoro Alyson E. (2018). Identifying protist consumers of photosynthetic picoeukaryotes in the surface ocean using stable isotope probing. Environmental Microbiology.

[23] Coskun OK, Pichler M, Vargas S, Gilder S, Orsi WD. Linking uncultivated microbial populations with benthic carbon turnover using quantitative stable isotope probing. Appl Environ Microbiol. 2018;84:e01083–18. 10.1128/AEM.01083-18 PMC6122004 29980553

[24] Dunford EA, Neufeld JD. DNA stable-isotope probing (DNA-SIP). J Vis Exp. 2010. 10.3791/2027. 10.3791/2027 PMC3156007 20729803

[25] Parada AE, Needham DM, Fuhrman JA (2016). Every base matters: assessing small subunit rRNA primers for marine microbiomes with mock communities, time series and global field samples. Environ Microbiol.

[26] Jochum LM, Chen X, Lever MA, Loy A, Jørgensen BB, Schramm A (2017). Depth distribution and assembly of sulfate-reducing microbial communities in marine sediments of Aarhus Bay. Appl Environ Microbiol.

[27] Rotthauwe JH, Witzel KP, Liesack W (1997). The ammonia monooxygenase structural gene amoA as a functional marker: molecular fine-scale analysis of natural ammonia-oxidizing populations. Appl Environ Microbiol.

[28] Geets J, Borremans B, Diels L, Springael D, Vangronsveld J, van der Lelie D (2006). DsrB gene-based DGGE for community and diversity surveys of sulfate-reducing bacteria. J Microbiol Methods.

[29] Müller AL, Kjeldsen KU, Rattei T, Pester M, Loy A (2015). Phylogenetic and environmental diversity of DsrAB-type dissimilatory (bi)sulfite reductases. ISME J.

[30] Pichler Monica, Coskun Ömer K., Ortega‐Arbulú Ana‐Sofia, Conci Nicola, Wörheide Gert, Vargas Sergio, Orsi William D. (2018). A 16S rRNA gene sequencing and analysis protocol for the Illumina MiniSeq platform. MicrobiologyOpen.

[31] Edgar RC (2010). Search and clustering orders of magnitude faster than BLAST. Bioinformatics.

[32] Edgar RC (2013). UPARSE: highly accurate OTU sequences from microbial amplicon reads. Nat Methods.

[33] Caporaso JG, Kuczynski J, Stombaugh J, Bittinger K, Bushman FD, Costello EK (2010). QIIME allows analysis of high-throughput community sequencing data. Nat Methods.

[34] Quast C, Pruesse E, Yilmaz P, Gerken J, Schweer T, Yarza P (2013). The SILVA ribosomal RNA gene database project: improved data processing and web-based tools. Nucleic Acids Res.

[35] Orsi WD, Smith JM, Liu S, Liu Z, Sakamoto CM, Wilken S (2016). Diverse, uncultivated bacteria and archaea underlying the cycling of dissolved protein in the ocean. ISME J.

[36] Youngblut ND, Barnett SE, Buckley DH (2018). HTSSIP: an R package for analysis of high throughput sequencing data from nucleic acid stable isotope probing (SIP) experiments. PLoS ONE.

[37] Gouy M, Guindon S, Gascuel O (2010). SeaView version 4: a multiplatform graphical user interface for sequence alignment and phylogenetic tree building. Mol Biol Evol.

[38] Edgar RC (2004). MUSCLE: multiple sequence alignment with high accuracy and high throughput. Nucleic Acids Res.

[39] Guindon S, Dufayard JF, Lefort V, Anisimova M, Hordijk W, Gascuel O (2010). New algorithms and methods to estimate maximum-likelihood phylogenies: assessing the performance of PhyML 3.0. Syst Biol.

[40] Rice P, Longden I, Bleasby A (2000). EMBOSS: the European Molecular Biology Open Software Suite. Trends Genet.

[41] Trifinopoulos J, Nguyen LT, von Haeseler A, Minh BQ (2016). W-IQ-TREE: a fast online phylogenetic tool for maximum likelihood analysis. Nucleic Acids Res.

[42] Kalyaanamoorthy S, Minh BQ, Wong TKF, von Haeseler A, Jermiin LS (2017). ModelFinder: fast model selection for accurate phylogenetic estimates. Nat Methods.

[43] Letunic I, Bork P (2016). Interactive tree of life (iTOL)v3: an online tool for the display and annotation of phylogenetic and other trees. Nucleic Acids Res.

[44] Team R. (2015). RStudio: integrated development for R.

[45] Blomberg SP, Garland T, Ives AR (2003). Testing for phylogenetic signal in comparative data: behavioral traits are more labile. Evolution.

[46] Pagel M (1999). Inferring the historical patterns of biological evolution. Nature.

[47] Keck F, Rimet F, Bouchez A, Franc A (2016). phylosignal: an R package to measure, test, and explore the phylogenetic signal. Ecol Evol.

[48] Morrissey EM, Mau RL, Schwartz E, Caporaso JG, Dijkstra P, van Gestel N (2016). Phylogenetic organization of bacterial activity. ISME J.

[49] Morrissey EM, Mau RL, Schwartz E, Koch BJ, Hayer M, Hungate BA (2018). Taxonomic patterns in the nitrogen assimilation of soil prokaryotes. Environ Microbiol.

[50] Morrissey EM, Mau RL, Schwartz E, McHugh TA, Dijkstra P, Koch BJ, et al. Bacterial carbon use plasticity, phylogenetic diversity and the priming of soil organic matter. ISME J. 2017. 10.1038/ismej.2017.43 PMC5520031 28387774

[51] Koch BJ, McHugh TA, Hayer M, Schwartz E, Blazewicz SJ, Dijkstra P (2018). Estimating taxon-specific population dynamics in diverse microbial communities. Ecosphere.

[52] Lueders T, McGenity TJ, Timmis KN, Nogales B (2015). DNA- and RNA-based stable isotope probing of hydrocarbon degraders. Hydrocarbon and lipid microbiology protocols: genetic, genomic and system analyses of communities..

[53] Dhillon A, Teske A, Dillon J, Stahl DA, Sogin ML (2003). Molecular characterization of sulfate-reducing bacteria in the Guaymas Basin. Appl Environ Microbiol.

[54] Schmidtko S, Stramma L, Visbeck M (2017). Decline in global oceanic oxygen content during the past five decades. Nature.

[55] Wright JJ, Konwar KM, Hallam SJ (2012). Microbial ecology of expanding oxygen minimum zones. Nat Rev Microbiol.

[56] D’Avanzo C, Kremer JN (1994). Diel oxygen dynamics and anoxic events in an eutrophic estuary of Waquoit Bay, Massachusetts. Estuaries.

[57] Kemp WM, Boynton WR, Adolf JE, Boesch DF, Boicourt WC, Brush G (2005). Eutrophication of Chesapeake Bay: historical trends and ecological interactions. Mar Ecol Prog Ser.

[58] Lavik G, Stührmann T, Brüchert V, Van der Plas A, Mohrholz V, Lam P (2009). Detoxification of sulphidic African shelf waters by blooming chemolithotrophs. Nature.

[59] Orsi W (2018). Ecology and evolution of seafloor and subseafloor microbial communities. Nat Rev Microbiol.

[60] Rickard D, Luther GW (2007). Chemistry of iron sulfides. Chem Rev.

[61] Jørgensen BB (1977). Bacterial sulfate reduction within reduced microniches of oxidized marine sediments. Mar Biol.

[62] Rabus R, Venceslau SS, Wohlbrand L, Voordouw G, Wall JD, Pereira IA (2015). A post-genomic view of the ecophysiology, catabolism and biotechnological relevance of sulphate-reducing prokaryotes. Adv Microb Physiol.

[63] Giuffre A, Borisov VB, Arese M, Sarti P, Forte E (2014). Cytochrome bd oxidase and bacterial tolerance to oxidative and nitrosative stress. Biochim Biophys Acta.

[64] Jia B, Jeong HI, Kim KH, Jeon CO (2016). Complete genome of Zhongshania aliphaticivorans SM-2(T), an aliphatic hydrocarbon-degrading bacterium isolated from tidal flat sediment. J Biotechnol.

[65] Dyksma S, Bischof K, Fuchs BM, Hoffmann K, Meier D, Meyerdierks A (2016). Ubiquitous Gammaproteobacteria dominate dark carbon fixation in coastal sediments. ISME J.

[66] Garcia R, Müller R, Rosenberg E, DeLong EF, Lory S, Stackebrandt E, Thompson F (2014). The family Polyangiaceae. The prokaryotes: deltaproteobacteria and epsilonproteobacteria..

[67] Youssef NH, Farag IF, Rinke C, Hallam SJ, Woyke T, Elshahed MS (2015). In silico analysis of the metabolic potential and niche specialization of candidate phylum “Latescibacteria” (WS3). PLoS ONE.

[68] Wasmund K, Mußmann M, Loy A (2017). The life sulfuric: microbial ecology of sulfur cycling in marine sediments. Environ Microbiol Rep.

[69] Anantharaman K, Hausmann B, Jungbluth SP, Kantor RS, Lavy A, Warren LA (2018). Expanded diversity of microbial groups that shape the dissimilatory sulfur cycle. ISME J.

[70] Hausmann B, Pelikan C, Herbold CW, Köstlbacher S, Albertsen M, Eichorst SA (2018). Peatland Acidobacteria with a dissimilatory sulfur metabolism. ISME J.

[71] Pester M, Bittner N, Deevong P, Wagner M, Loy AA (2010). ‘Rare biosphere’ microorganism contributes to sulfate reduction in a peatland. ISME J.

[72] Hoehler TM, Jørgensen BB (2013). Microbial life under extreme energy limitation. Nat Rev Microbiol.

[73] Hausmann B, Pelikan C, Rattei T, Loy A, Pester M. Long-term transcriptional activity at zero growth by a cosmopolitan rare biosphere member. *bioRxiv*. 2018. 10.1128/mBio.02189-18 PMC6372793 30755506

[74] Ye Q, Wu Y, Zhu Z, Wang X, Li Z, Zhang J (2016). Bacterial diversity in the surface sediments of the hypoxic zone near the Changjiang Estuary and in the East China Sea. Microbiologyopen.

[75] Ravenschlag K, Sahm K, Pernthaler J, Amann R (1999). High bacterial diversity in permanently cold marine sediments. Appl Environ Microbiol.

[76] Ghai R, Mizuno CM, Picazo A, Camacho A, Rodriguez-Valera F (2013). Metagenomics uncovers a new group of low GC and ultra-small marine Actinobacteria. Sci Rep.

[77] Rosenberg E, Dworkin M, Falkow S, Rosenberg E, Schleifer KH, Stackebrandt E (2006). Hydrocarbon-oxidizing bacteria. The prokaryotes: volume 2: ecophysiology and biochemistry.

[78] Inagaki F, Suzuki M, Takai K, Oida H, Sakamoto T, Aoki K (2003). Microbial communities associated with geological horizons in coastal subseafloor sediments from the sea of okhotsk. Appl Environ Microbiol.

[79] Cornall A, Rose A, Streten C, McGuinness K, Parry D, Gibb K (2016). Molecular screening of microbial communities for candidate indicators of multiple metal impacts in marine sediments from northern Australia. Environ Toxicol Chem.

[80] Nogales B, Moore ER, Llobet-Brossa E, Rossello-Mora R, Amann R, Timmis KN (2001). Combined use of 16S ribosomal DNA and 16S rRNA to study the bacterial community of polychlorinated biphenyl-polluted soil. Appl Environ Microbiol.

[81] Camanocha Anuj, Dewhirst Floyd E. (2014). Host-associated bacterial taxa from Chlorobi, Chloroflexi, GN02, Synergistetes, SR1, TM7, and WPS-2 Phyla/candidate divisions. Journal of Oral Microbiology.

[82] Dyksma S, Lenk S, Sawicka JE, Mußmann M (2018). Uncultured gammaproteobacteria and desulfobacteraceae account for major acetate assimilation in a coastal marine sediment. Front Microbiol.

[83] Dyksma S, Pjevac P, Ovanesov K, Mussmann M (2018). Evidence for H2 consumption by uncultured Desulfobacterales in coastal sediments. Environ Microbiol.

[84] Szewzyk R, Pfennig N (1987). Complete oxidation of catechol by the strictly anaerobic sulfate-reducing Desulfobacterium catecholicum sp. nov. Arch Microbiol.

[85] Phelps CD, Kerkhof LJ, Young LY (1998). Molecular characterization of a sulfate-reducing consortium which mineralizes benzene. FEMS Microbiol Ecol.

