# Supplementary material for: Quantifying population-specific growth in benthic bacterial communities under low oxygen using H218O
Source: ISME J. 2019 Feb 19;13(6):1546–59. doi: 10.1038/s41396-019-0373-4 (PMC6776007; doi:10.1038/s41396-019-0373-4)
Supplement: Supplementary file 1 — Fig S1 [file 41396_2019_373_MOESM1_ESM.pdf]

Quantifying population-specific activities in benthic bacterial communities under low oxygen using $H_2^{18}O$

Ömer K. Coskun[1], Volkan Özen[1], Scott D. Wankel[3], and William D. Orsi[1,2§]

Supplemental Information

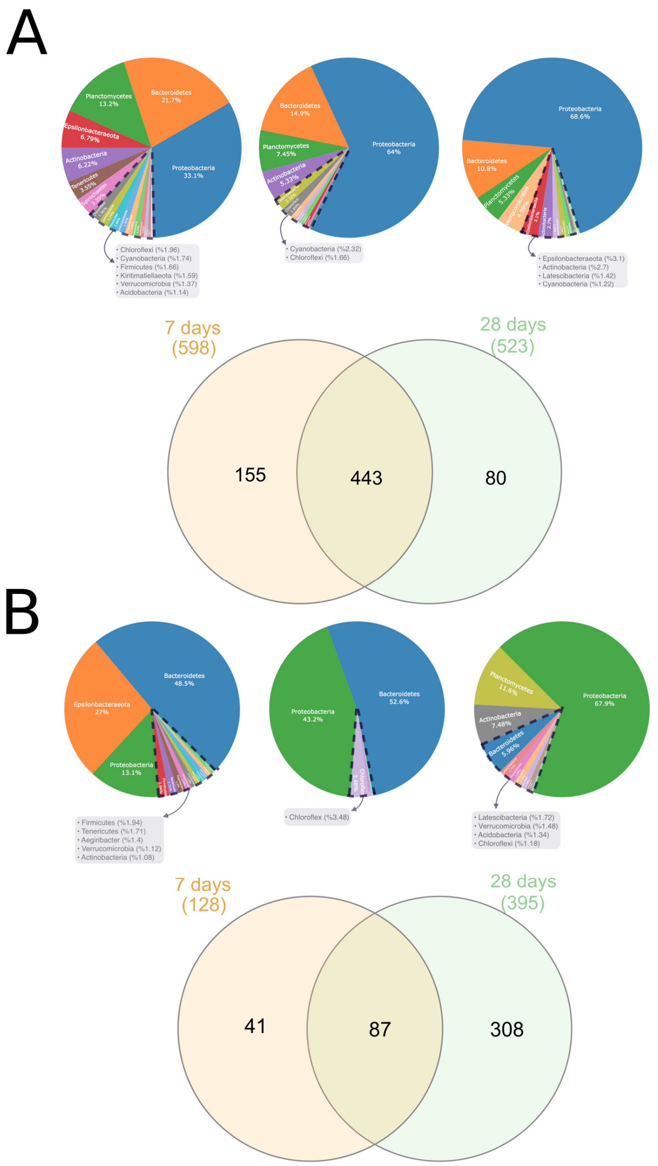

Figure S1: Pie charts displaying the proportions (%) and phylum-level composition of total (A) and labeled (B) 16S rRNA gene sequences together with corresponding number of overlapping and non-overlapping OTUs between the experimental timepoints.
